# *cis*-Bis{(*E*)-2-[(2-fluoro­phen­yl)imino­meth­yl]phenolato-κ^2^ *N*,*O*}bis­(pyridine-κ*N*)nickel(II)^1^

**DOI:** 10.1107/S1600536812045576

**Published:** 2012-11-10

**Authors:** Marcela C. G. Souza, Leonardo da C. Ferreira, Nadia M. Comerlato, Glaucio B. Ferreira, Lorenzo do Canto Visentin

**Affiliations:** a Instituto de Química, Universidade Federal do Rio de Janeiro, 21949-900 Rio de Janeiro, RJ, Brazil; b Instituto de Química, Departamento de Química Inorgânica, Universidade Federal Fluminense, 24020-150 Niterói, RJ, Brazil; c R&D NanoBusiness, e-Diffraction Pharma, 22451-900 Rio de Janeiro, RJ, Brazil

## Abstract

The structure of the title compound, [Ni(C_13_H_9_FNO)_2_(C_5_H_5_N)_2_], consists of an Ni^II^ atom on a crystallographic center of symmetry, octa­hedrally bonded through both the N and O atoms to two 2-[(2-fluoro­phen­yl)imino­meth­yl]phenolate (*L*) ligands, as well as two pyridine ligands. The F atoms of *L* are disordered over two positions related by a 180° rotation of the fluoro­phenyl group around its external C—N bond.

## Related literature

For related nickel compounds, see: Dang *et al.* (2009 ▶); Orpen *et al.* (1989 ▶). For related *N*-salicyl­idene anilines, see: Lindeman *et al.* (1981 ▶); Temel *et al.* (2007 ▶); Çelik *et al.* (2009 ▶).
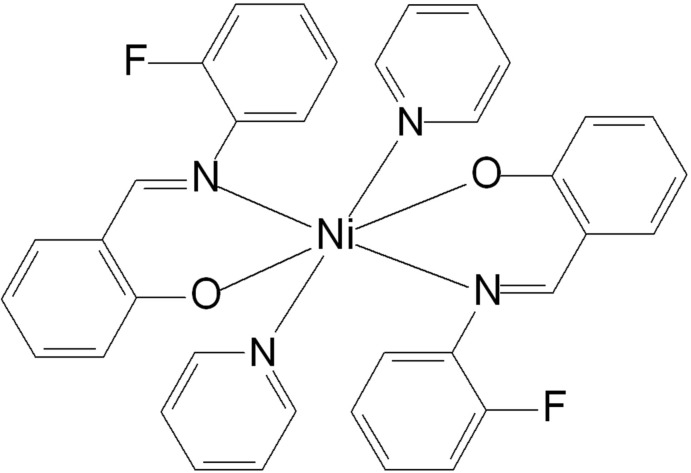


## Experimental

### 

#### Crystal data

[Ni(C_13_H_9_FNO)_2_(C_5_H_5_N)_2_]
*M* *_r_* = 645.32
Triclinic, 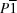

*a* = 8.3160 (17) Å
*b* = 10.344 (2) Å
*c* = 11.049 (2) Å
α = 109.94 (3)°
β = 98.53 (3)°
γ = 111.93 (3)°
*V* = 786.2 (3) Å^3^
*Z* = 1
Mo *K*α radiation
μ = 0.67 mm^−1^
*T* = 295 K
0.17 × 0.16 × 0.12 mm

#### Data collection

Nonius KappaCCD diffractometer
Absorption correction: multi-scan (*SADABS*; Sheldrick, 2004 ▶) *T*_min_ = 0.895, *T*_max_ = 0.924
11574 measured reflections
2909 independent reflections
2652 reflections with *I* > 2σ(*I*)
*R*_int_ = 0.026

#### Refinement

*R*[*F* ^2^ > 2σ(*F* ^2^)] = 0.037
*wR*(*F* ^2^) = 0.105
*S* = 1.05
2909 reflections
219 parameters
H atoms treated by a mixture of independent and constrained refinement
Δρ_max_ = 0.41 e Å^−3^
Δρ_min_ = −0.32 e Å^−3^

### 

Data collection: *COLLECT* (Hooft, 1998 ▶); cell refinement: *PHICHI* (Duisenberg *et al.*, 2000 ▶); data reduction: *EVALCCD* (Duisenberg *et al.*, 2003 ▶); program(s) used to solve structure: *SHELXS97* (Sheldrick, 2008 ▶); program(s) used to refine structure: *SHELXL97* (Sheldrick, 2008 ▶); molecular graphics: *ORTEP-3 for Windows* (Farrugia, 2012 ▶); software used to prepare material for publication: *WinGX* (Farrugia, 2012 ▶).

## Supplementary Material

Crystal structure: contains datablock(s) I, global. DOI: 10.1107/S1600536812045576/br2213sup1.cif

Structure factors: contains datablock(s) I. DOI: 10.1107/S1600536812045576/br2213Isup2.hkl

Additional supplementary materials:  crystallographic information; 3D view; checkCIF report

## Figures and Tables

**Fig. 1.**
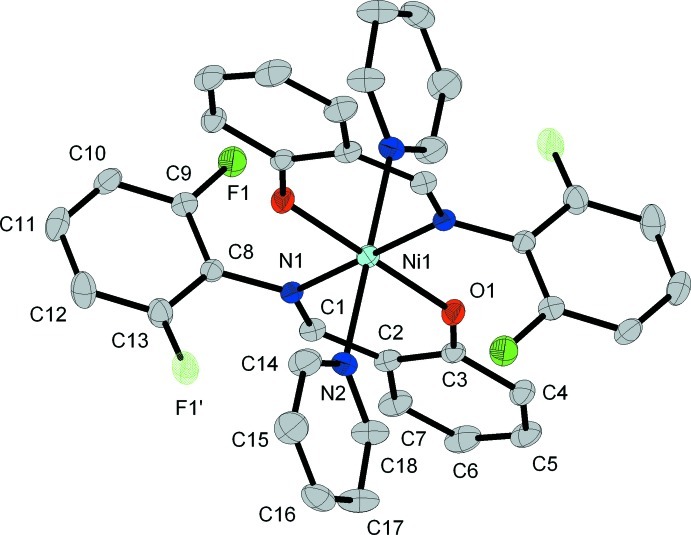
ORTEP projection of the title molecule. Thermal ellipsoids are at the 30% probability level.

## supplementary crystallographic information

### Comment

N-Salicylidene anilines (Lindeman *et al.*, 1981; Temel *et al.*, 2007; Çelik *et al.*, 2009;) are Schiff bases easily prepared by condensation of salicylaldehyde and an aniline. These molecules act as ligands with transition metals. The Ni^II^ cation is octahedrally coordinated by four N and two O atoms with only slight distortion from the ideal coordination geometry. The two independent 2-(*E*)-(2-fluorophenyliminomethyl)phenolato ligands are bidentate and provide each one N atom from the imine moiety and one phenolato O atom. The coordination is completed by the two pyridine N atoms in trans arrangement. The Ni—N and Ni—O distances (Table 1) are in the typical ranges and like all other interatomic distances are in good agreement with literature data (Dang, *et al.*, 2009; Orpen *et al.*, 1989). The dihedral angle between C8-C13 and C2-C7 phenyl rings is 82.9 (1)°, This compound exhibits a statistical disorder, showing two partial fluorine atoms in unequal proportions (0.700 (4):0.300 (4)), F1 and F1', respectively.

### Experimental

For 2-fluorophenyliminomethyl)phenol ligand (LH). To a solution of 2-hydroxybenzaldehyde (2.6 ml, 25.0 mmol) in 50.0 ml of ethanol was added a mixture of NaOH/HCl (3.0 ml, pH 5.0) and 2-fluoroaniline (2.4 ml, 25.0 mmol). The reaction mixture was kept under reflux for 2 h with continuous stirring. The reaction mixture was cooled to 273 K for 24 h, the yellow precipitate was collected and washed with water and ethanol. Yield: 70%. M.P. 328–330 K; Elemental Analysis, CHN, for C_13_H_10_F_1_N_1_O_1_ (Found C, 78.63; H, 5.62; N, 6.96. Calculate: C, 79.16; H, 6.37;N, 7.10%).

For C_36_H_26_F_2_N_4_O_2_Ni. A solution of LH (0.430 g, 0.2 mmol), 3.0 ml of MeOH / MeONa (10% *w*/*v*) and 5.0 ml of pyridine in 20.0 ml of acetone was stirred for five minutes. Then nickel(II) acetate (0.176 g, 0.1 mmol) was added. The reaction mixture was stirred for 24 h at room temperature. The solution was filtered off and red block crystals suitable for X-ray analysis were obtained by slow evaporation of the solution at room temperature. Yield: 55%. M.P. 569–571 K; Elemental Analysis, CHN, for C_36_H_26_F_2_N_4_O_2_Ni (Found C, 67.86; H,4.15; N, 7.38. Calculated: C, 68.39; H, 4.45; N, 8.14%).

### Refinement

H atoms of the unsaturated carbon were positioned geometrically (C–H = 0.93 Å for Csp2 atoms) and treated as riding on their respective C atoms, with *U*_iso_(H) values set at 1.2UeqCsp2. The H9 and H13 atoms were omitted for solve of the disorder in the F1 and F1' atoms attached in C9 and C13.

### Crystal data

**Table:**

[Ni(C_13_H_9_FNO)_2_(C_5_H_5_N)_2_]	*Z* = 1
*M**_r_* = 645.32	*F*(000) = 332
Triclinic, *P*1	*D*_x_ = 1.359 Mg m^−^^3^
Hall symbol: -P 1	Melting point: 569 K
*a* = 8.3160 (17) Å	Mo *K*α radiation, λ = 0.71073 Å
*b* = 10.344 (2) Å	Cell parameters from 370 reflections
*c* = 11.049 (2) Å	θ = 1–27.5°
α = 109.94 (3)°	µ = 0.67 mm^−^^1^
β = 98.53 (3)°	*T* = 295 K
γ = 111.93 (3)°	Block, red
*V* = 786.2 (3) Å^3^	0.17 × 0.16 × 0.12 mm

### Data collection

**Table:**

Nonius KappaCCD diffractometer	2909 independent reflections
Radiation source: fine-focus sealed tube	2652 reflections with *I* > 2σ(*I*)
Graphite monochromator	*R*_int_ = 0.026
φ scans, and ω scans with κ	θ_max_ = 25.5°, θ_min_ = 3.8°
Absorption correction: multi-scan (*SADABS*; Sheldrick, 2004)	*h* = −10→9
*T*_min_ = 0.895, *T*_max_ = 0.924	*k* = −12→12
11574 measured reflections	*l* = −13→13

### Refinement

**Table:**

Refinement on *F*^2^	Primary atom site location: structure-invariant direct methods
Least-squares matrix: full	Secondary atom site location: difference Fourier map
*R*[*F*^2^ > 2σ(*F*^2^)] = 0.037	Hydrogen site location: inferred from neighbouring sites
*wR*(*F*^2^) = 0.105	H atoms treated by a mixture of independent and constrained refinement
*S* = 1.05	*w* = 1/[σ^2^(*F*_o_^2^) + (0.0566*P*)^2^ + 0.389*P*] where *P* = (*F*_o_^2^ + 2*F*_c_^2^)/3
2909 reflections	(Δ/σ)_max_ < 0.001
219 parameters	Δρ_max_ = 0.41 e Å^−^^3^
0 restraints	Δρ_min_ = −0.32 e Å^−^^3^

### Special details

**Table:**

Geometry. All e.s.d.'s (except the e.s.d. in the dihedral angle between two l.s. planes) are estimated using the full covariance matrix. The cell e.s.d.'s are taken into account individually in the estimation of e.s.d.'s in distances, angles and torsion angles; correlations between e.s.d.'s in cell parameters are only used when they are defined by crystal symmetry. An approximate (isotropic) treatment of cell e.s.d.'s is used for estimating e.s.d.'s involving l.s. planes.
Refinement. Refinement of *F*^2^ against ALL reflections. The weighted *R*-factor *wR* and goodness of fit *S* are based on *F*^2^, conventional *R*-factors *R* are based on *F*, with *F* set to zero for negative *F*^2^. The threshold expression of *F*^2^ > σ(*F*^2^) is used only for calculating *R*-factors(gt) *etc*. and is not relevant to the choice of reflections for refinement. *R*-factors based on *F*^2^ are statistically about twice as large as those based on *F*, and *R*- factors based on ALL data will be even larger.

### Fractional atomic coordinates and isotropic or equivalent isotropic displacement parameters (Å^2^)

**Table:**

	*x*	*y*	*z*	*U*_iso_*/*U*_eq_	Occ. (<1)
Ni1	0.0000	0.0000	0.0000	0.04073 (15)	
C1	0.3568 (3)	0.1876 (2)	−0.0377 (2)	0.0434 (5)	
C2	0.4086 (3)	0.3195 (2)	0.0890 (2)	0.0438 (5)	
C3	0.3036 (3)	0.3221 (2)	0.1802 (2)	0.0419 (5)	
C4	0.3826 (4)	0.4568 (3)	0.3068 (2)	0.0563 (6)	
H4	0.3208	0.4613	0.3706	0.068*	
C5	0.5489 (4)	0.5805 (3)	0.3367 (3)	0.0677 (8)	
H5	0.5966	0.6664	0.4200	0.081*	
C6	0.6465 (4)	0.5792 (3)	0.2444 (3)	0.0700 (8)	
H6	0.7565	0.6642	0.2644	0.084*	
C7	0.5771 (3)	0.4504 (3)	0.1237 (3)	0.0598 (6)	
H7	0.6427	0.4486	0.0624	0.072*	
C8	0.1974 (3)	−0.0607 (2)	−0.2117 (2)	0.0388 (4)	
C9	0.1232 (3)	−0.0664 (3)	−0.3351 (2)	0.0513 (5)	
C10	0.0995 (4)	−0.1814 (3)	−0.4576 (2)	0.0645 (7)	
H10	0.0511	−0.1816	−0.5392	0.077*	
C11	0.1480 (4)	−0.2945 (3)	−0.4574 (3)	0.0661 (7)	
H11	0.1311	−0.3727	−0.5389	0.079*	
C12	0.2215 (5)	−0.2914 (3)	−0.3360 (3)	0.0705 (8)	
H12	0.2560	−0.3671	−0.3356	0.085*	
C13	0.2449 (4)	−0.1764 (3)	−0.2140 (3)	0.0574 (6)	
C14	0.0620 (5)	−0.2436 (3)	0.0879 (3)	0.0752 (8)	
H14	−0.0445	−0.3115	0.0149	0.090*	
C15	0.1386 (6)	−0.3036 (4)	0.1603 (4)	0.0860 (10)	
H15	0.0836	−0.4091	0.1363	0.103*	
C16	0.2949 (4)	−0.2069 (4)	0.2667 (3)	0.0716 (8)	
H16	0.3489	−0.2448	0.3167	0.086*	
C17	0.3709 (4)	−0.0527 (4)	0.2986 (3)	0.0793 (9)	
H17	0.4783	0.0167	0.3704	0.095*	
C18	0.2847 (4)	−0.0020 (3)	0.2217 (3)	0.0674 (7)	
H18	0.3368	0.1034	0.2450	0.081*	
N1	0.2146 (2)	0.05438 (19)	−0.08618 (16)	0.0381 (4)	
N2	0.1329 (2)	−0.0937 (2)	0.11714 (19)	0.0463 (4)	
O1	0.1449 (2)	0.21035 (17)	0.15535 (15)	0.0486 (4)	
H1	0.438 (4)	0.204 (3)	−0.085 (3)	0.060 (7)*	
F1	0.0721 (4)	0.0386 (3)	−0.3371 (2)	0.0771 (9)	0.700 (4)
F1'	0.2941 (10)	−0.1824 (8)	−0.1059 (5)	0.086 (2)	0.300 (4)

### Atomic displacement parameters (Å^2^)

**Table:**

	*U*^11^	*U*^22^	*U*^33^	*U*^12^	*U*^13^	*U*^23^
Ni1	0.0372 (2)	0.0367 (2)	0.0399 (2)	0.01420 (16)	0.00849 (15)	0.01184 (16)
C1	0.0392 (11)	0.0459 (11)	0.0413 (11)	0.0147 (9)	0.0096 (9)	0.0210 (9)
C2	0.0423 (11)	0.0365 (10)	0.0426 (11)	0.0118 (9)	0.0013 (9)	0.0181 (9)
C3	0.0453 (11)	0.0330 (9)	0.0389 (10)	0.0185 (9)	−0.0003 (8)	0.0113 (8)
C4	0.0629 (15)	0.0420 (12)	0.0468 (12)	0.0257 (11)	0.0013 (11)	0.0051 (10)
C5	0.0727 (17)	0.0331 (11)	0.0590 (15)	0.0178 (12)	−0.0163 (13)	0.0004 (11)
C6	0.0599 (15)	0.0407 (13)	0.0728 (17)	0.0015 (11)	−0.0091 (14)	0.0206 (12)
C7	0.0515 (14)	0.0476 (13)	0.0606 (15)	0.0063 (11)	0.0030 (11)	0.0258 (11)
C8	0.0333 (9)	0.0387 (10)	0.0382 (10)	0.0125 (8)	0.0109 (8)	0.0144 (8)
C9	0.0509 (13)	0.0586 (13)	0.0438 (12)	0.0271 (11)	0.0104 (10)	0.0208 (10)
C10	0.0603 (15)	0.0769 (18)	0.0370 (12)	0.0270 (14)	0.0055 (11)	0.0124 (11)
C11	0.0730 (17)	0.0494 (13)	0.0513 (14)	0.0183 (13)	0.0222 (13)	0.0045 (11)
C12	0.101 (2)	0.0541 (15)	0.0755 (18)	0.0470 (16)	0.0434 (17)	0.0295 (13)
C13	0.0748 (17)	0.0597 (14)	0.0545 (14)	0.0419 (13)	0.0264 (12)	0.0282 (12)
C14	0.090 (2)	0.0529 (15)	0.0689 (17)	0.0363 (15)	−0.0046 (15)	0.0190 (13)
C15	0.117 (3)	0.0642 (18)	0.083 (2)	0.0542 (19)	0.012 (2)	0.0334 (16)
C16	0.0747 (18)	0.103 (2)	0.0810 (19)	0.0589 (18)	0.0324 (16)	0.0635 (18)
C17	0.0539 (15)	0.097 (2)	0.086 (2)	0.0232 (15)	0.0016 (14)	0.0591 (19)
C18	0.0504 (14)	0.0648 (16)	0.0787 (18)	0.0138 (12)	0.0018 (13)	0.0435 (14)
N1	0.0367 (8)	0.0374 (8)	0.0353 (8)	0.0162 (7)	0.0070 (7)	0.0130 (7)
N2	0.0430 (10)	0.0518 (10)	0.0495 (10)	0.0241 (8)	0.0144 (8)	0.0252 (8)
O1	0.0457 (8)	0.0416 (8)	0.0439 (8)	0.0158 (7)	0.0116 (6)	0.0081 (6)
F1	0.120 (2)	0.0914 (17)	0.0525 (13)	0.0771 (17)	0.0231 (13)	0.0362 (12)
F1'	0.143 (6)	0.110 (5)	0.060 (3)	0.104 (5)	0.038 (3)	0.045 (3)

### Geometric parameters (Å, º)

**Table:**

N1—Ni1	2.128 (2)	C8—N1	1.431 (3)
N2—Ni1	2.251 (2)	C9—C10	1.390 (3)
Ni1—O1^i^	2.003 (2)	C10—C11	1.373 (4)
Ni1—O1	2.003 (2)	C10—H10	0.9300
Ni1—N1^i^	2.128 (2)	C11—C12	1.374 (4)
Ni1—N2^i^	2.251 (2)	C11—H11	0.9300
C1—N1	1.294 (3)	C12—C13	1.388 (4)
C1—C2	1.444 (3)	C12—H12	0.9300
C1—H1	0.92 (3)	C14—N2	1.333 (3)
C2—C7	1.419 (3)	C14—C15	1.383 (4)
C2—C3	1.428 (3)	C14—H14	0.9300
C3—O1	1.300 (3)	C15—C16	1.359 (5)
C3—C4	1.430 (3)	C15—H15	0.9300
C4—C5	1.384 (4)	C16—C17	1.366 (4)
C4—H4	0.9300	C16—H16	0.9300
C5—C6	1.394 (5)	C17—C18	1.384 (4)
C5—H5	0.9300	C17—H17	0.9300
C6—C7	1.368 (4)	C18—N2	1.324 (3)
C6—H6	0.9300	C18—H18	0.9300
C7—H7	0.9300	F1—C9	1.311 (3)
C8—C9	1.382 (3)	F1'—C13	1.234 (5)
C8—C13	1.387 (3)		
			
O1—Ni1—N1	88.26 (7)	C4—C5—C6	121.4 (2)
O1^i^—Ni1—N1	91.74 (7)	C4—C5—H5	119.3
O1—Ni1—N1^i^	91.74 (7)	C6—C5—H5	119.3
O1^i^—Ni1—N1^i^	88.26 (7)	C5—C4—C3	121.5 (3)
O1—Ni1—N2	89.03 (7)	C5—C4—H4	119.3
O1^i^—Ni1—N2	90.97 (7)	C3—C4—H4	119.3
N1—Ni1—N2	91.69 (7)	C11—C12—C13	120.6 (3)
N1^i^—Ni1—N2	88.31 (7)	C11—C12—H12	119.7
O1—Ni1—N2^i^	90.97 (7)	C13—C12—H12	119.7
O1^i^—Ni1—N2^i^	89.03 (7)	C11—C10—C9	119.6 (2)
N1—Ni1—N2^i^	88.31 (7)	C11—C10—H10	120.2
N1^i^—Ni1—N2^i^	91.69 (7)	C9—C10—H10	120.2
C3—O1—Ni1	131.3 (1)	N2—C18—C17	124.2 (3)
C1—N1—C8	116.6 (2)	N2—C18—H18	117.9
C1—N1—Ni1	125.0 (2)	C17—C18—H18	117.9
C8—N1—Ni1	118.3 (1)	C10—C11—C12	119.5 (2)
C18—N2—C14	115.9 (2)	C10—C11—H11	120.2
C18—N2—Ni1	121.7 (2)	C12—C11—H11	120.2
C14—N2—Ni1	122.3 (2)	C6—C7—C2	122.2 (3)
C9—C8—C13	117.5 (2)	C6—C7—H7	118.9
C9—C8—N1	121.6 (2)	C2—C7—H7	118.9
C13—C8—N1	120.9 (2)	C7—C6—C5	118.7 (2)
N1—C1—C2	127.1 (2)	C7—C6—H6	120.7
N1—C1—H1	120 (2)	C5—C6—H6	120.7
C2—C1—H1	113 (2)	N2—C14—C15	123.4 (3)
O1—C3—C2	124.2 (2)	N2—C14—H14	118.3
O1—C3—C4	119.2 (2)	C15—C14—H14	118.3
C2—C3—C4	116.6 (2)	C15—C16—C17	118.4 (3)
C7—C2—C3	119.6 (2)	C15—C16—H16	120.8
C7—C2—C1	116.6 (2)	C17—C16—H16	120.8
C3—C2—C1	123.8 (2)	C16—C17—C18	118.6 (3)
F1—C9—C8	119.2 (2)	C16—C17—H17	120.7
F1—C9—C10	118.9 (2)	C18—C17—H17	120.7
C8—C9—C10	121.9 (2)	C16—C15—C14	119.4 (3)
F1'—C13—C8	118.5 (3)	C16—C15—H15	120.3
F1'—C13—C12	120.4 (3)	C14—C15—H15	120.3
C8—C13—C12	120.9 (2)		
			
C8—N1—C1—C2	−177.3 (2)		

Symmetry code: (i) −*x*, −*y*, −*z*.
